# Thermal effects of monopolar electrosurgery detected by real-time infrared thermography: an experimental appendectomy study

**DOI:** 10.1186/s12893-020-00735-6

**Published:** 2020-05-27

**Authors:** Taras V. Nechay, Svetlana M. Titkova, Mikhail V. Anurov, Elena V. Mikhalchik, Kirill Y. Melnikov-Makarchyk, Ekaterina A. Ivanova, Alexander E. Tyagunov, Abe Fingerhut, Alexander V. Sazhin

**Affiliations:** 1 Pirogov Russian National Research Medical University, Ostrovitianov str. 1, Moscow, 117997 Russia; 2 Research and Clinical Center for Physical-Chemical Medicine, Malaya Pirogovskaya 1a, Moscow, 119435 Russia; 3 Section for Surgical Research, Department of Surgery, Medical University of Graz, 8036 Graz, Austria; 4 Department of Gastrointestinal Surgery, Ruijin Hospital, Shanghai Jiao Tong University School of Medicine, Shanghai, 20025 China

**Keywords:** Appendectomy, Monopolar electrosurgery, Complications after appendectomy, Thermography, Lateral thermal spread

## Abstract

**Background:**

Monopolar energy (ME) is routinely used in appendectomy. This study aimed to investigate the degree of lateral thermal spread generated by ME and to evaluate the thermal injury sustained by the close-lying tissues.

**Methods:**

Appendectomy with a monopolar Maryland dissector was performed in 8 rabbits (at 30 and 60 W power settings). A high-resolution infrared camera was used to record tissue heating during the intervention. After autopsy macroscopic changes were evaluated and tissue samples were subjected to myeloperoxidase (MPO) assay and histological examination.

**Results:**

No significant differences in the extent of thermal spread, MPO activity and histological signs of inflammation were observed between groups. Regardless of the power settings, the heat spread exceeded 2 cm laterally along the mesoappendix when application time exceeded 3 s. The spread of heat through tubular structures in both groups caused a significant temperature rise in the nearby intestinal loop, resulting in perforation (*n* = 3) and necrosis (*n* = 1).

**Conclusions:**

Application time is critical in thermal spread during appendectomy aided by ME. Tubular anatomic structures can enhance thermal injury on distant tissues. The observed effects of ME bear clinical relevance that need further investigation.

## Background

Electrosurgical techniques for tissue dissection and hemostasis have long become an essential component of surgical routine. Of 230 million surgical interventions performed globally every year, the majority rely heavily on electrical energy [1, 2]. Both patients and surgeons benefit from electrosurgery since it significantly increases the cutting speed and improves the quality of hemostasis. Conversely, it also can cause complications. In spite of the continuous evolution of the technology, the incidence of serious adverse events during or following electrosurgery (2–5 for 1000 patients)***,*** shows no tendency to decline [1, 3]. Most often, such complications arise from monopolar energy and account for 53% of all undesirable effects associated with electrosurgery [1]. Apart from direct injury, the dangers of monopolar electrosurgery include residual heat injury, direct coupling, capacitive coupling, and antenna coupling [4], all of which can be potentially traumatic for a patient under certain conditions [5, 6]. These effects are well described with the goal of avoiding high-risk scenarios for surgical energy-based device-related complications. Several resources are available regarding this topic, including The Society of American Gastrointestinal and Endoscopic Surgeons (SAGES), Fundamental Use of Surgical Energy (FUSE) program, and others. Despite this, numerous surveys of surgeons show significant knowledge gaps in the safe use of widely used energy-based devices [7].

Thus, Malik has found a “high level of ignorance regarding the procedure and indications of basic electrosurgical equipment” [8]. Feldman shows lack of knowledge in basic electrosurgical principles among members of SAGES board having discovered only 59% percent of correct answers on the safe use of electrosurgery test among them [9]. Despite advances in electrosurgical devices, a monopolar electrosurgical unit invented back in 1926 by William T. Bovie remains one of the most available and widely utilized surgical instruments [10].

Inadvertent damage and delayed injury to nearby tissue resulting from the lateral spread of thermal energy can be sustained during any type of electrosurgery. The degree of thermal spread depends on the type of instrument used, its power settings and application time [11]. Monopolar energies cause a rise in temperature to a potentially critical level at a 1-cm distance from the top of an electrosurgical instrument even when the applied power is low [12]. Moreover, monopolar diathermy generates greater lateral thermal spread than more advanced electrosurgical instruments used for dissection and hemostasis during laparoscopic appendectomy [11]. Borie described 876 complications of surgical energy-driven devices for 7 years, most of them (70%) caused by monopolar energy. The main reason, highlighted by author was “persistent misunderstanding of appropriate usage within the medical and paramedical teams” [13]. Most experimental and clinical studies have evaluated the degree of lateral thermal spread at high or maximum electrosurgical unit (ESU) power settings by a histological examination [14]. However, there were certain limitations to the experimental design of these studies: they were conducted in vitro using bovine and porcine muscle samples, whose overall resistance differed from the resistance of the intestinal wall and the mesoappendix in vivo. Besides, the lack of blood supply may have affected thermal spread in the samples.

Laparoscopic appendectomy is a common surgical procedure that often involves the use of a monopolar electrosurgical energy [15]. According to the World Society of Emergency Surgery (WSES), monopolar electrocoagulation and bipolar energy are “the most cost-effective techniques for mesoappendix dissection, even if more experience and technical skills are required to avoid potential complications and thermal injuries” [16].

As lateral thermal damage is more pronounced in patients with an inflamed appendix [17, 18], and drawing on the literature [12], we hypothesized that lateral thermal spread would be greater with higher power settings and that the heating of nearby tissue, including the cecum and the mesoappendix, could lead to complications. The aim of this study was to investigate the lateral thermal spread during monopolar-aided appendectomy at different ESU power settings using real-time infrared thermography and to evaluate the severity of thermal injury to the affected tissue.

## Methods

### Animals

The experiment was conducted in 8 male Soviet chinchilla rabbits weighing from 3770 to 4240 g (mean weight was 3981 ± 174 g) obtained from University vivarium. The animals were kept in separate cages under standard conditions at 20–22 °C, 40–50% humidity and 12:12 h light/dark regime with free access to food and water. Before surgery, the animals were allowed to acclimate for 7 days. All animal studies were performed following the ARRIVE guidelines and conducted in strict accordance with the Guide for Care and Use of Laboratory Animals, Pirogov Russian National Research Medical University, Moscow.

### Power sources

In this work, we used the Alsa EXCELL NHP 250/D ESU with a constant voltage and power output, equipped with start pulse and high frequency leakage controls. Cutting and coagulation were performed in the Pure (non-modulated sinusoidal current for the cut without any coagulating effect) and Soft (modulated low-voltage current with a strong deep effect and no superficial carbonization) modes, respectively.

### The experiment

The rabbits were fasted for 12 h prior to surgery but had free access to water. The surgical procedures were performed under general anesthesia with an intravenous injection of ketamine (Ketamin 10%, Sanofi-Ceva, Dusseldorf, Germany) and xylazine (Rompun 2%, Bayer, Leverkusen, Germany) under sterile conditions. The animals were placed on a surgical table in a supine position. The skin was shaved, cleaned and sterilized, and an 8-cm long midline laparotomy was performed. The appendix was delivered into the wound. Appendectomy was carried out following the standard technique: the mesoappendix was separated from the appendix from tip to base using a 5 mm monopolar Maryland dissector with operating part 16 mm × 2 mm for each branch (KELLY dissecting and grasping forceps, Maryland, Karl Storz, Germany) connected to the ESU. The size of the mesenteric tissue grasped by the dissector did not exceed 4 mm. The animals were distributed into two groups depending on the applied power mode: cutting and coagulation at 30 W (LP group) AND cutting and coagulation at 60 W (SP group). The tissue was desiccated as opposed to fulguration, i.e. the electrode was in direct contact with the tissue. The duration of a single application was not strictly limited and depended on the time required to achieve the desired effect. We only counted the number of applications and their total time. The unburied stump was ligated with Polyglycolide 3/0 sutures. After appendectomy, the abdominal wound was closed by sutures. The animals were monitored after surgery and brought back to the vivarium as soon as they awoke from anesthesia. The animals were allowed to drink water on the day of surgery; solid food was introduced on the following day.

### Intraoperative thermography

Real-time thermography was performed using the science grade FLIR A655sc camera with 30 Hz frequency, thermal sensitivity of < 50 mK and a resolution of 640 × 480 pixels. The camera was mounted 1 m above the surgical site.

The obtained thermal images of the cecum, where tissue was later collected for further histological examination and the enzyme assay, were processed in FLIR ResearchIR MAX Version 4.40.6.24. We also investigated how heat spread from the mesoappendix to the adjacent organs and noted the time required for tissue cooling. The critical threshold temperature for cell damage was assumed to be 42 °C. All surgical interventions were recorded on a high-definition video camera (Canon 7D) mounted on a tripod at the same angle and distance as the infrared camera; the recording was synchronized with thermography.

### Euthanasia of research animals

On day 5, the animals were anesthetized and then sacrificed with an overdose of barbiturates (Narcoren®, 100 mg/kg, intravenous). During the necropsy, we looked for the visible signs of intraperitoneal inflammation and tissue adhesions in the peritoneal cavity. The serosal surface of the cecum wall was examined for any signs of perforation, edema or discoloration. To assess the severity of tissue adhesions, a modified Vetere grading scale [19] was used: 0 points – no adhesions, 1 point – thin, easily separable adhesions, 2 points – thick, difficult to separate adhesions.

### Histological analysis

**Fig. 1:**
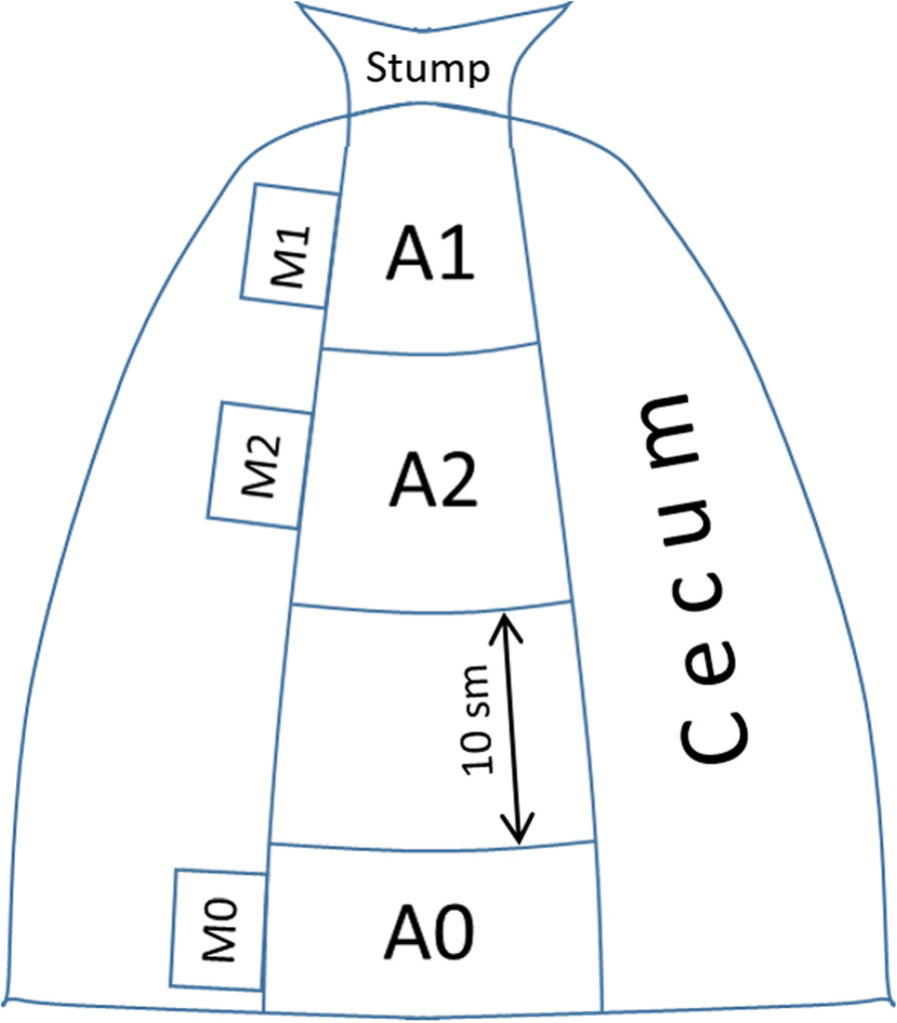
A schematic representation of material sampling for histological analysis (A0-A2) and measurement of MPO activity in tissue samples (M0-M2)

All samples were collected after euthanasia of the animals. The schematic representation of sample collection for the histological analysis is presented in Fig. 1.

Two areas of the cecum sized 1х1 cm (А1, adjacent to the stump, and А2, laterally and adjacent to A1) were excised during the necropsy and fixed in formalin for 24 h. Sample A0 excised from a region of the large intestine lying at a distance exceeding the potential spread of heat (10 cm) from the surgical site was used as a reference. The collected samples were paraffinized and later cut into 5-μm slices. The obtained tissue sections were stained with hematoxylin-eosin and Van Gieson stain to assess thermal damage to collagen fibers. Standard light microscopy was performed using a Motic BA-400 Trinocular microscope (Motic China Group Co., Ltd., China). The images were processed in Motic Images Advanced 3.2 software. The severity and extent of cell infiltration and tissue edema were calculated from the micrographs. All samples were studied by a histologist blinded to the experiment.

### Tissue myeloperoxidase assay

Samples M1 and M2 were collected from the regions adjacent to A1 and A2 and tested for myeloperoxidase (MPO) levels. A tissue region distant to the cecum was used as a reference (Fig. 1). MPO activity was measured in the samples of the intestinal wall. Briefly, the frozen samples of the intestinal tissue were homogenized in 0.01 M PBS (pH 7.4) containing 0.2% Triton X-100 using a Potter-Elvehjem tissue grinder. The homogenates were centrifuged at 900 g for 30 min. Then, equal volumes of 1% hexadecyl trimethyl ammonium chloride solution in PBS were added to each supernatant, and MPO activity was determined. The supernatant (50 μL) was mixed with 150 μL of 0.1 M sodium citrate buffer (pH 5.5) containing 0.1 mg/mL^− 1^ of O-dianisidine chloride and 0.001% H_2_O_2_ used as a substrate for MPO activity. After incubating the mixture for 5 min, the reaction was stopped with 1 ml of 35% orthophosphoric acid. Absorbance was measured at 460 nm. MPO activity was calculated based on the extinction coefficient of 20,040 M^−1^cm^− 1^7. The Lowry assay was used to quantitate the total protein content in the supernatants. MPO activity was expressed as nM/mg of protein.

All animals were weighed before operation and on post-operative day 5. No antimicrobial prophylaxis was subscribed. No additional parameters of animals recovery were assessed.

### Statistical analysis

The obtained data was processed in Statistica 13.3 for Windows (StatSoft Inc., Tulsa, OK, USA). The results were presented as mean values and standard deviations (SD). The Student’s t- test was applied to compare continuous variables. The Mann-Whitney U-test was used to compare nonparametric quantitative data. The normality of data distribution was analyzed using Kolmogorov–Smirnov and Shapiro–Wilk tests. Differences were considered significant at *p* < 0.05. A power analysis was not applicable because of the exploratory nature of the study and because it was part of a subproject of a larger study.

## Results

**Fig. 2:**
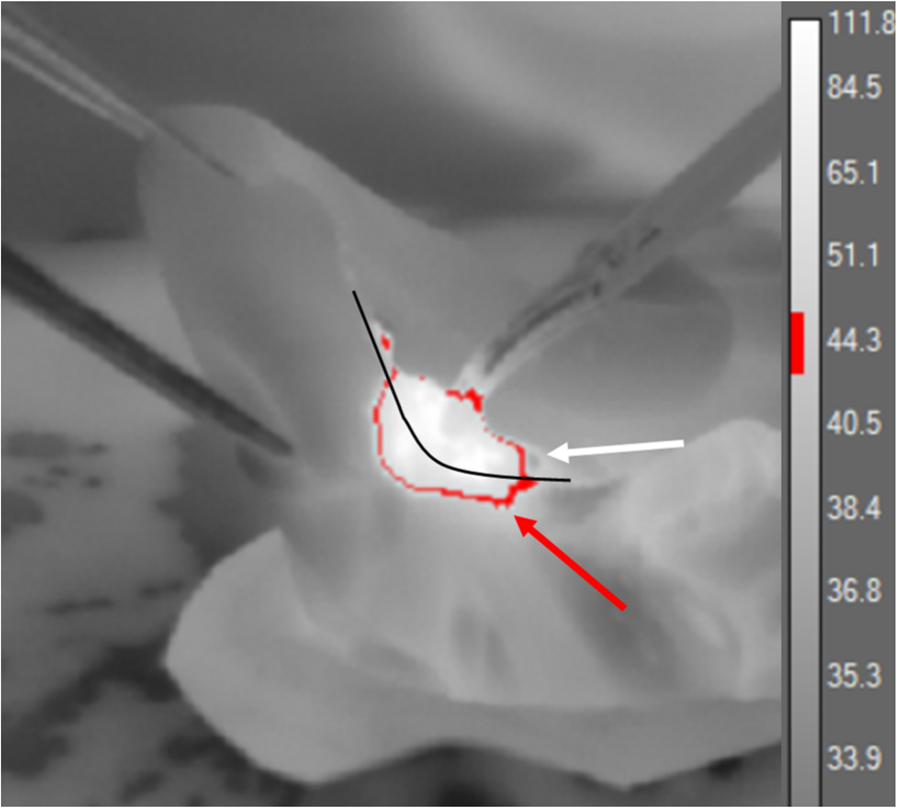
A thermogram of the cecum (to the left of the black line) during mesoappendix (to the right of the black line) dissection. The temperature is given in degrees Celsius. Inside the red zone, the temperature is over 45 °C; outside the red zone, it is below 42 °C; at the border, the temperature is 42–45 °C

The appendix size (length and width) was similar in both groups: 9.44 (1.02) cm and 0.95 (0.13) cm in LP and 10.06 (0.86) cm and 1.02 (0.24) cm in SP, respectively. Ambient temperature was 20 °C. The total number of analyzed applications in our experiment was 704; total application time was 203 (39) s in LP and 118 (18) s in SP (*р* = 0.008). At low power, the average duration of a single application was 2.05 (0.53) s, whereas at standard power settings, it took 1.34 (0.22) s to attain satisfactory hemostasis or good quality of cut (*р* = 0.112). Maximal duration of a single application reached 9.7 (1.5) s in LP group and 5.03 (1.55) s in SP group (*p* = 0.03). The number of applications was the same in both experimental groups. Extensive lateral thermal spread during electrocoagulation was observed in all animals. Regardless of the power settings, the heat spread more than 2 cm laterally along the mesoappendix when application time exceeded 3 s. No significant differences in the extent of thermal spread were observed for low and standard power settings. The highest temperature of the cecum recorded during the surgery was 61.3 °C (Fig. 2).

The cecum temperature increased when the mesoappendix coagulation site was 1 cm away from the cecum, correlating with coagulation time and showing no dependency on power settings.

It took 12 to 42 s for the cecum to cool down to the point below 42 °C, the critical cell damage temperature cutoff used in this study. The analysis of thermal images revealed that intense heating of the mesoappendiceal fat during the dissection of the mesoappendix caused a temperature rise in the cecal wall as late as 3–5 s following the application of monopolar energy. It took 30 s for the mesoappendix heated over 100 °C during dissection to cool down to 42 °C. The cooling pattern was not linear: the higher the temperature of the heated tissue in comparison with the temperatures of the animal’s body and the environment, the faster the cooling rate, approximately 10 degrees per second. When the tissue cooled down to 60–70 °C, the cooling rate dropped to 10 degrees per 10 s. Thermography data are presented in Table 1.

**Table 1:** Thermography data

Group	Low Power					Standard Power					
	R1	R3	R6	R8	Mean (SD)	R2	R4	R5	R7	Mean (SD)	p
Mean application time, s	2.5	1.46	2.5	1.75	2.05 (0.53)	1.2	1.09	1.52	1.53	1.34 (0.22)	0.112
Min application time, s	0.3	0.1	0.4	0.5	0.33 (0.17)	0.1	0.4	0.4	0.2	0.28 (0.15)	0.773
Max application time, s	11.5	7.9	9.8	9.6	9.7 (1.5)	4.1	3.4	6.8	5.8	5.03 (1.55)	0.03
Application duration > 3 s, %	n/d*	6,3%	25%	6,8%	12.7 (10.7)	3%	1,2%	11,8%	10,3%	6.6 (5.3)	0.595
Cecum T max, °C	58.9	55.3	59.1	48.4	55.4 (4.9)	61.3	57.2	48.8	56.1	55.9 (5.2)	0.885
Mesoappendix T max °C	152.4	146	160.4	157.2	154 (6.3)	160.6	156.4	160.8	160.5	159.6 (2.1)	0.112
T ≥ 42° duration on cecum^a^, s	42	n/d^b^	13.4	15.5	23.6 (15.9)	24	14	12	13	15.8 (5.6)	0.377
Lateral thermal spread^a^, mm	8	n/d^b^	4.6	4.48	5.7 (1.9)	5.6	5.6	6	9.3	6.6 (1.8)	0.372
Lateral thermal spread on mesoappendix^a^, mm	28	24	27.6	20.4	25 (3.6)	33	18.4	20.4	32.4	26.1 (7.7)	0.886
Lateral thermal spread on intestinal loop, mm	1.8	1.6	2.5	3.3	2.3 (0.8)	1.4	1.6	3	6	3 (2.1)	0.886
Complication	no	no	Intestinal wall full thickness necrosis	Intestinal perforation	2/4	no	no	Intestinal perforation	Intestinal perforation	2/4	

^a^ ≥42 °C

^b^no data

Weight measurements on postoperative day 5 revealed that the animals had lost 268 (53) g on average, but the differences between the groups were not statistically significant. During the necropsy, we observed no visible macroscopic changes in the cecal serosa and noticed no signs of hyperemia, necrosis or perforation of the cecum. The peritoneal cavity showed no signs of pathological fluid buildup. Adhesions had started to form at the site of the surgical scar in one animal from the SP group and in 2 animals from the LP group, involving the loops of the large or small intestines (adhesion severity scored 1 point on the Vetere scale).

**Fig. 3:**
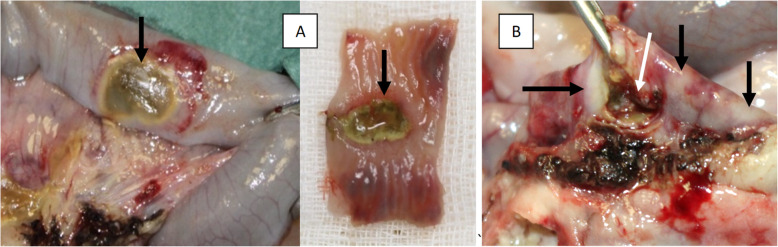
Damage to the intestinal loop caused by thermal energy. **a** zones of transmural coagulation necrosis without perforation (black arrows). **b** perforation of the small intestine wall (a white arrow) and a few areas of thermal damage to the serosa

One rabbit from the LP group developed full-thickness necrosis of the intestinal wall (1х1 cm) without any signs of perforation (Fig. 3a, marked by a black arrow). Two rabbits from the SP group and one rabbit from the LP group had perforation of the small intestine wall (Fig. 3b, marked by a white arrow). Close to the perforation site, there were a few areas of coagulative necrosis in the intestinal wall with no signs of perforation (Fig. 3b, marked by black arrows). There was also a soft, loose adhesion between the perforated region and the loop of the small intestine. Dissection of the adhesion provoked leakage of the intestinal contents into the peritoneal cavity.

### Histological examination

Minimal to moderate infiltration with plasma cells, lymphocytes, and eosinophils was observed in all layers of the cecal wall 1 and 2 cm away from the stump in both groups on day 5 after appendectomy. The mucosa, submucosa and subserosa were edematous to various degrees (Table 2).

**Table 2:** Pathologic findings at the different sites of the cecum layers on day 5 after appendectomy using monopolar electrocoagulation

Power	30 W		60 W	
Distance from stump	1 cm	2 cm	1 cm	2 cm
**Cell infiltration**				
**Severity**				
Absent Moderate Marked	2/4 (50%) 1/4 (25%) 1/4 (25%)	0/4 2/4 (50%) 2/4 (50%)	2/4 (50%) 2/4 (50%)	2/4 (50%) 1/4 (25%) 1/4 (25%)
Minimal: < 10%	0/4	0/4	0/4	1/4 (25%)
Mild: 10–25%;	1/4 (25%)	0/4	1/4 (25%)	1/4 (25%)
Moderate: 26–50%	2/4 (50%)	3/4 (75%)	3/4 (75%)	2/4 (50%)
Marked: > 51%	1/4 (25%)	1/4 (25%)	0/4	0/4
**Extent**				
Mucosal	2/4 (50%)	2/4 (50%)	2/4 (50%)	3/4 (75%)
Mucosal and submucosal	1/4 (25%)	1/4 (25%)	1/4 (25%)	0/4
Mucosal, submucosal and subserosal	1/4 (25%)	1/4 (25%)	1/4 (25%)	1/4 (25%)
**Edema**				
**Severity**				
Absent Moderate Marked	2/4 (50%) 1/4 (25%) 1/4 (25%)	0/4 2/4 (50%) 2/4 (50%)	2/4 (50%) 2/4 (50%)	2/4 (50%) 1/4 (25%) 1/4 (25%)
**Extent** Submucosal Mucosal and submucosal Mucosal, submucosal and subserosal	1/4 (25%) 1/4 (25%)	4/4 (100%)	1/4 (25%) 1/4 (25%)	2/4 (50%)

**Fig. 4:**
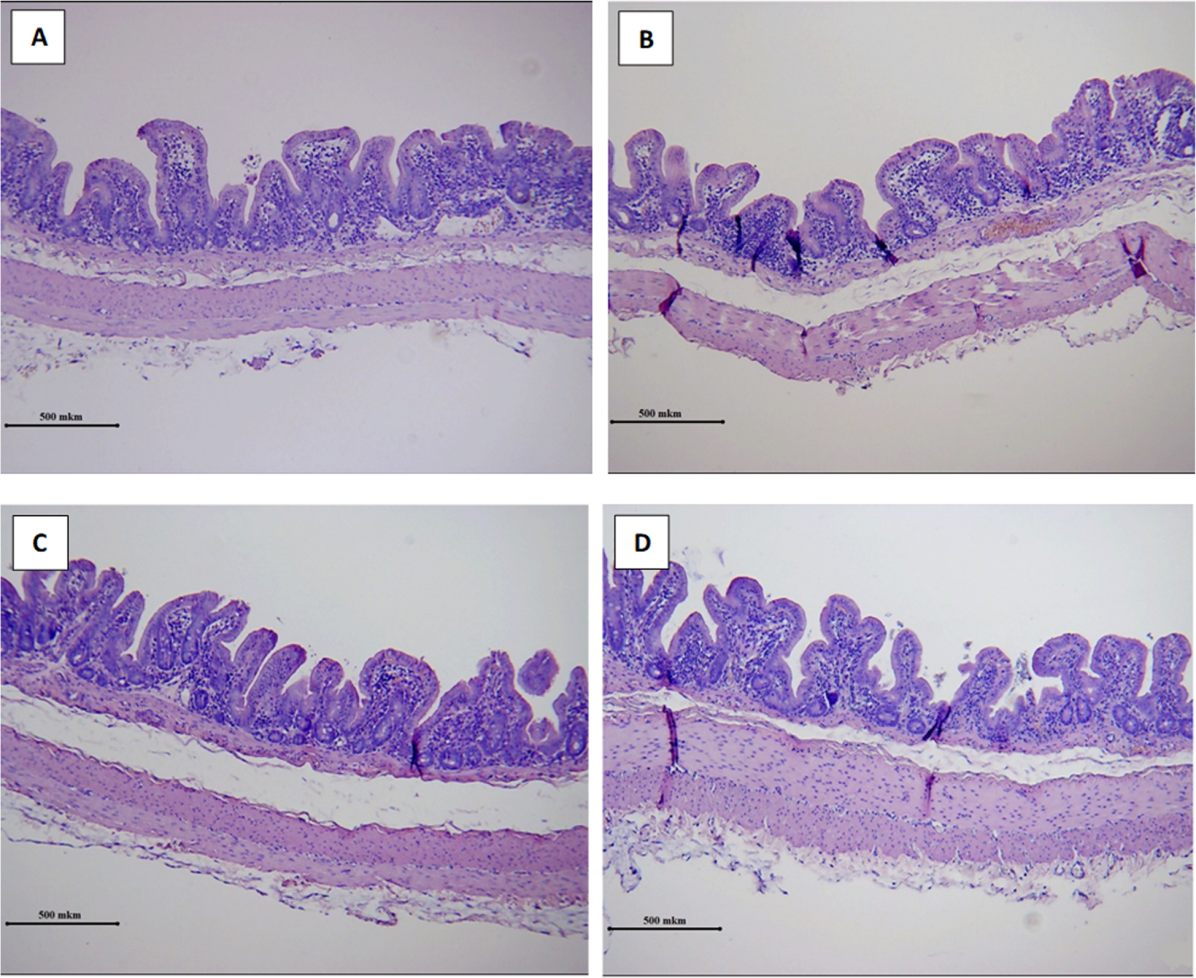
Representative images of H&E-stained cecum sections illustrate histopathological changes on day 5 following appendectomy aided by monopolar surgical energy. **a** (LP group) and **c** (SP group) - 1 cm from the stump; **b** (LP group) and **d** (SP group) - 2 cm from the stump (× 100, scale bar 500 μm)

The muscular layer fibers exhibited no signs of swelling or degeneration. No damage to collagen fibers or neutrophil infiltration was noticed in the samples (Fig. 4). One rabbit from the LP group had an extensive hemorrhagic lesion in the muscular layer, another one had a small area of subepithelial necrosis 1 cm from the stump. In one rabbit from the SP group, a large mucosal erosion was observed 2 cm from the stump.

### Tissue myeloperoxidase

**Fig. 5:**
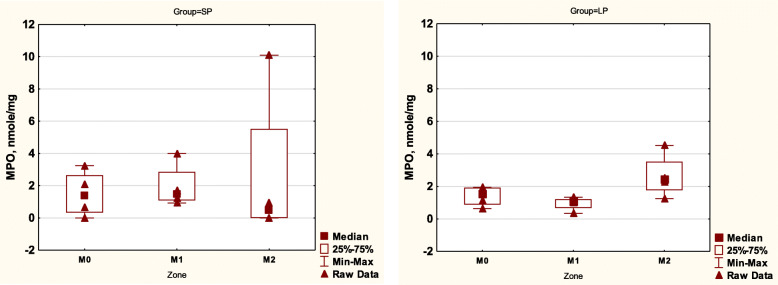
MPO activity in tissue samples. R – animals, high – ME 60 W, low – 30 W

The enzyme assay did not detect any significant differences between tissue myeloperoxidase levels in the studied samples and the reference values (Fig. 5). The differences between the groups were not statistically significant.

### Effects of monopolar coagulation during appendectomy

**Fig. 6:**
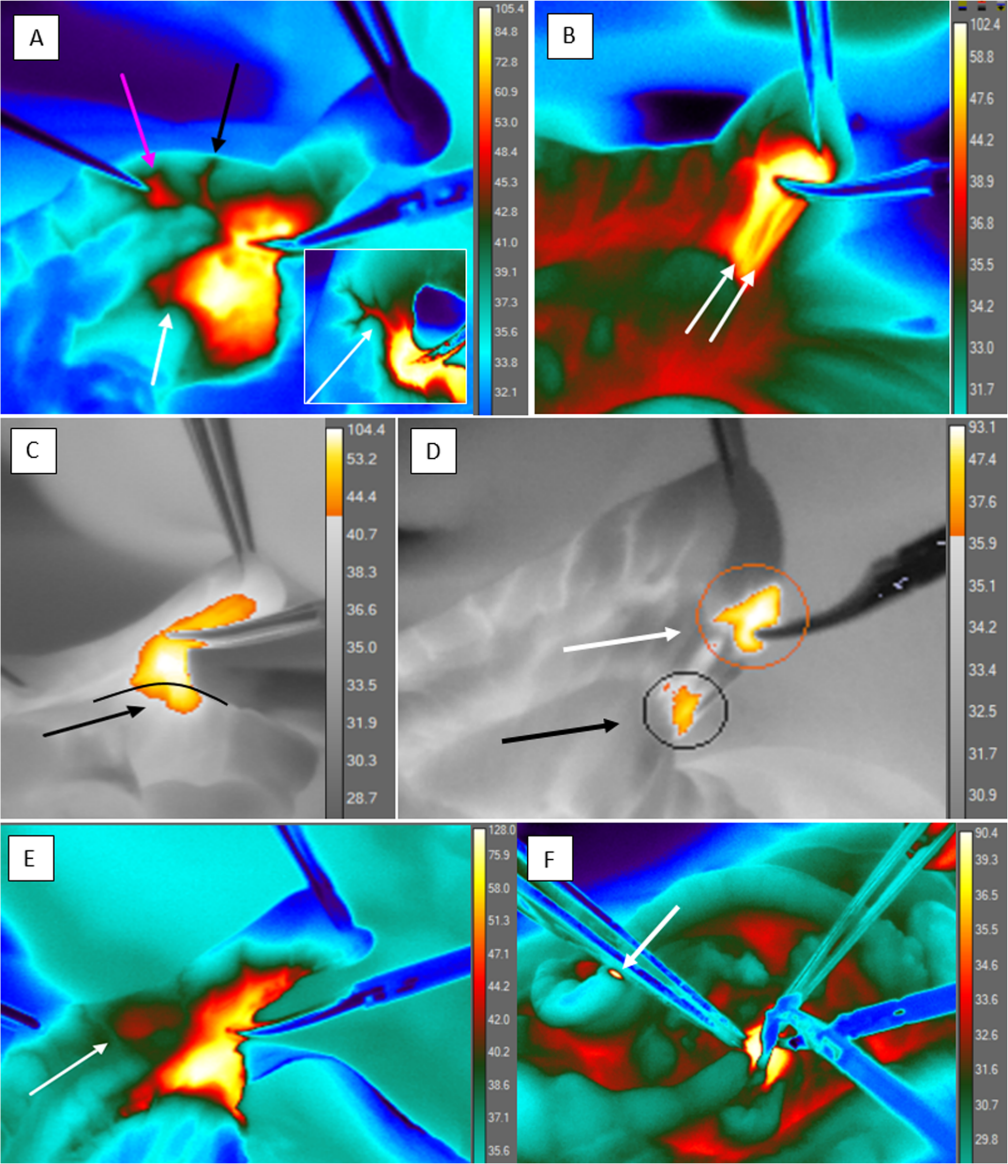
Effects of ME in appendectomy. **a** Current channeling (a black arrow), the “clamp effect” (a purple arrow), mesenteric vessel cooling by blood flow (a white arrow). **b** Current channeling (white arrows). **c** Lateral thermal spread causes heating of the surrounding tissue (a small intestine loop – a black arrow). **d** The “pedicle effect” causes heating of tissues where a smaller tubular structure (a vessel – a white arrow) enters a larger tubular structure (an intestinal loop – a black arrow). **e** The “jump over” effect (a white arrow). **f** Current leakage through the nonactive instrument (a white arrow)

The thermal images obtained during the surgery were retrospectively analyzed in-depth and compared to necropsy reports. The following thermal effects were noticed:

- The heat spread through tubular structures (Fig. 6a, marked by a black arrow; Fig. 6b). Passing through the mesenteric vessels, it caused a temperature rise in the small intestine (Fig. 6c), resulting in the perforation of the intestinal wall in three rabbits in both groups (Fig. 3b) and transmural necrosis without wall perforation in one more rabbit (Fig. 3a);
- The mesenteric tissue was cooled by the large vessels passing through its thickness and supplying blood to the small intestinal loop adjacent to the appendix. (Fig. 6a, marked by a white arrow). The blood vessels heated up almost instantly and gave off a considerable amount of heat to the surrounding tissue; they also cooled down quickly, but the mesoappendix tissue stayed heated for a longer time;
- The “clamp effect”: the tissue held with the forceps or clamp jaws was heating up as the electrical current generated by ME was flowing through the forceps due to the high current density in the grasped tissue (Fig. 6a, marked by a purple arrow).
- The “pedicle effect”: the heating of tissue (orange circle) along the tubular structures (the mesenteric vessels) in the area where such structure (artery marked by a white arrow) entered a larger tubular structure (an intestinal loop; marked by a black arrow) (Fig. 6d).
- The “jump over” effect (Fig. 6e, marked by a white arrow).
- Leakage of the electrical current through the nonactive instrument when it accidentally touched the loop of the small intestine (Fig. 6f, a white arrow).

Analysis of thermograms showed that one to three of the above-mentioned effects were detected in each of animal with perforation or necrosis. With that, the lateral thermal spread on intestinal loop in these animals was significantly greater than in animals without complications (3.7 (1.6) mm vs.1.6 (0.16) mm, *p* = 0.029). In animals with complications average percent of applications ≥3 s was 13.5 (7.9) vs. 3.5 (2.6) in animals without complications (*p* = 0.052).

## Discussion

Regarding WSES Jerusalem guidelines for treatment of acute appendicitis no difference in several methods of mesoappendix dissection was reported. But stating the danger of leaving foreign bodies in the abdominal cavity, utilization of energy devices was preferable over clipping of appendicular artery. Moreover guidelines suggest energy devices in case of inflamed and oedematous mesoappendix consider ME as “the most costeffective method” among them [16]. According to the study of J R. Robinson et al. [20] surgeons were informed about cost-reducing opportunities in a real-time while making a decision of taking a preferrable device for mesoappendix dissection. This led monopolar hook to became the most often device for mesoappendix dissection (61.3%). Neither SAGES nor EAES guidelines on diagnosis and management of acute appendicitis provide information about preferable energy device for appendectomy [21, 22].

This study has shown that the duration of ME was the key factor affecting the extent of thermal spread during appendectomy, not the power. Regardless of the power settings (30 or 60 W), heat spread more than 2 cm laterally along the mesoappendix when application time exceeded 3 s. Perforation correlated with long-lasting single exposure both in LP and SP groups. In all animals with perforation a single exposure of monopolar energy lasted over 1.5 s thereby causing rising of the temperature of the small intestine to a critical threshold through the entire thickness of the intestinal wall (2 mm).

It is difficult to predict and measure thermal spread resulting from ME application [10]. Several authors [18] have hypothesized or reported that lower power settings would lead to less thermal spread. In fact, surgeons who use ME devices use different power settings, modes and activation times depending on the task [23]. To our knowledge, no standardized curriculum for surgeons has so far addressed the safe use of ME for the dissection of the mesoappendix during laparoscopic appendectomy. The majority of manuals recommend applying “the lowest possible power setting” [24], others suggest setting the power to 70 to 90 W in the “pure cutting” mode or to 50 W in the “coagulation” mode [25]. Jones et al., showed in a randomized clinical trial that thermal injury occurred more frequently in the coagulation mode compared to the blend mode at the level of the trocar incision sites [26]. The WSES, EAES and SAGES guidelines for diagnosis and treatment of acute appendicitis provide no recommendations regarding ESU power and voltage for appendectomy [16, 21, 22]. In our study, “coagulation” and “cutting” modes were fixed at 30 and 60 W because general surgeons tend to use such combinations [27] for the mesentery dissection. Besides, we aimed to compare results of our in vivo study with main ex vivo experimental and in vivo clinical studies of lateral thermal spread [11, 12, 28, 29].

Previous studies have demonstrated that the area of living tissue affected by a thermal burn gradually grows in size after the initial impact, reaching its maximum by days 3 to 7, and does not start to shrink until day 14 [18]. Damage to nearby structures typically occurs on day 4–5 following the surgery due to unrecognized energy transfer [25, 30]. Although the cecum had been heated up to 42 °C (“critical temperature”), no clinical manifestations were noticed on postoperative day 5. At the same time, histological examination revealed signs of moderate inflammation and reactive edema affecting all tissue layers both at a distance of 1 cm and 2 cm from the stump, attesting to thermal exposure. However, at the time of necropsy, the inflammation was aseptic in nature: there was no neutrophil infiltration and MPO levels in the cecal tissue were low. This could be explained by the design of our experiment: appendectomy was performed on the non-inflamed appendix. A study of lateral thermal damage to the mesoappendix and the appendiceal base during laparoscopic appendectomy in children demonstrated that the postoperative pain syndrome and the duration of hospital stay directly depend on the temperature of the heated tissue and the size of the thermal lesion [17].

Due to gaps in scientific data concerning critical level of temperature for creation of intestine damage we set the cut-off value for “critical temperature” for possible cell damage at 42 °C, as it was done by other investigators [6, 31]. Commonly it was based on a known fact that heated over 42 °C cell suffers from protein denaturation [32]. However, in fact, there is no consensus as to what temperature should be considered critical in terms of cell damage. Experimental studies have found that cells heated in excess of 20 °C die within 15 s; heated in excess of 25 °C, in 4 s, and heated in access of 30 °C, in 2 s [33]. We found no differences in the histopathological changes to the cecal tissue between the studied groups. Total ME application times and surgery duration were longer in the LP group than in the SP group. This could be explained by the fact that in order to achieve the same effect at low power settings, the duration of a single application needs to be increased. Longer application times lead to extensive lateral thermal spread; as a result, the tissues are exposed to the temperatures that cause cell damage for longer periods of time. This means that the severity of thermal injury does not change significantly when low power settings are applied, as compared to standard settings. The maximum temperature was the same at any power settings. In addition, three rabbits from both groups demonstrated a histological picture of thermal lesions in different layers of the cecal wall following the longest exposure of the cecum to T ≥ 42 °C. Previously, Hefermehl [12] conducted an experiment in order to investigate a relationship between thermal spread and power settings/application time. He discovered that at 60 W the area of thermal spread increased by one-third (in comparison with 30 W). Longer application times (2 s) led to a multifold increase in the lesion area from 3.5 to more than 20 mm. The majority of recommendations on the use of coagulation devices were developed based on ex vivo experiments on bovine and porcine musculofascial strips that had no blood flow in them [11, 12]. Results of our in vivo study carried out on organs with physiologically normal blood flow are for the most part in accordance with these data but demonstrate that the key factor leading to injury was duration of application. The overall percent of such “long-lasting” applications (≥3 s) in ¾ of animals with complications exceed 10%. This fact should be taken into account when elaborating surgical guidelines.

The dynamics of thermal spread may be influenced by the difference in blood supply to healthy and inflamed tissues or their hydrophilicity. However, Pogorelić reported no significant differences in lateral thermal spread during appendectomy in patients with acute appendicitis and in the absence of inflammation [17]. In patients with appendicitis, the diameter of the inflamed appendix changes along its course, which means that its regions can heat up to different temperatures as the current passes through it. In our experiment, the temperature of the appendix rose only after the heat reached the mesoappendix, and that rise was not very pronounced. We also observed the so-called “jump-over” effect indicating that thermal processes in deeper tissues can only be evaluated indirectly and sometimes can go unnoticed. Infrared camera captures a temperature rise in the serosa occurring after the appendix gets heated up through its entire thickness from inside. Probably the mesoappendix-appendix borderline played a role in preventing the appendix from heating. This observation calls into question the role of the appendix diameter in thermal spread.

In theory, thermal injury involves two major zones during appendectomy with ME: the mesoappendix and the cecum. Interestingly, however, three animals from both groups developed perforation of the small intestine, and another rabbit developed transmural necrosis of the small intestine. Based on the effects registered in our experimental study using thermal imaging, several possible underlying mechanisms might be assumed. First, current channeling through the mesenteric vessels might provoke substantial lateral thermal spread. Diamantis [18] compared the effects of different electrosurgical modalities (mono- and bipolar coagulation, impedance-controlled bipolar vessel sealing and US shears) on the short gastric vessels (1 mm in diameter) on rabbits. Four of 20 animals treated with ME developed perforation of the greater curvature of the stomach at the coagulation site on post-operative day 3. Bipolar coagulation resulted in stomach perforation in two rabbits (10%). Khan et al. [34] studied the effect of thermal spread on the prostatic nerve plexus during robotic prostatectomy in vivo. He demonstrated that interposition of the vessels significantly reduced thermal spread to distal tissues. In our case, the structures in the proximity to the coagulation site were affected because the appendix and the small intestinal loop share common blood vessels [35] and because heat travels through tubular structures rapidly. This effect was observed in all animals developed perforation or necrosis of small intestine.

Another possible cause of intestinal perforation is the pedicle effect described by Humes et al. [30]. This occurs when the electrical current goes through a tubular structure to the point where the latter enters another tubular structure of a larger diameter. Humes et al. demonstrated the pedicle effect in a clinical series of three patients with similar common bile duct perforations discovered during laparoscopic cholecystectomy and occurred at the junction of the cystic and common bile duct. A similar effect was observed in our study when the energy spread along the mesenteric vessels that entered the small intestinal loop. Our hypothesis would be that more heat was emitted at the vasculo-intestinal junction than along the vessels.

Another phenomenon observed in our study was the clamp effect (the heating of tissues grasped by forceps or a clamp through which the electrical current flows). It can be explained in terms of the physical fundamentals of electricity. When current passes through a conductor, it heats it up according to the Joule-Lenz law. As the conductor diameter decreases, resistance and local heat production increase. The clamp effect can be critical for tubular structures, such as the ureters or the intestinal wall, leading to their heating at a distance from the site of coagulation.

One to three of the above-mentioned effects were observed in each animal with perforation or necrosis of the small intestine. All effects were detected during retrospective analysis of thermograms, so we did not perform histological examination of the small intestinal loop in the proximity to the coagulation site in all animals. It is possible that other animals with registered effects had pathological changes of the intestinal wall, but not so pronounced.

Further detailed investigation is necessary to identify the conditions under which preformation can occur, to assess its severity and the risk of perforation when performing coagulation in proximity to tubular structures.

Because of inherent difficulties in retrospectively studying mechanisms of laparoscopic injuries, it is important to categorize potential mechanisms of unintended energy transfer in order to prevent bowel injuries [7].

Summing up, all of the listed effects may play a significant role in postoperative complications.

### Limitations of this study

We recognize that all laparoscopic appendectomies are not performed with ME, but this practice might be more prevalent than admitted. The major limitation of our study was that we used healthy animals with non-inflamed appendix, and therefore the potential enhancement of inflammation was not studied. No antimicrobial prophylaxis was administered to the animals in our study, so we do not know the effects of antibiotic on thermal spread. As heavy postoperative complications were not expected we didn’t plan to investigate the influence of antibiotics on their developing because it didn’t correlate with the purpose of the study. Another limitation of this study is the small sample size. Moreover we did not compare the effects of other hemostatic devices (bipolar, LigaSure, EnSeal, ultrasound etc). We recognize that while monopolar equipment is less expensive than the other modalities, there are ample data that show that ME might be more dangerous.

In addition, because of anatomical differences between species, the results obtained in this study might not be directly extrapolated to humans. Moreover, the duration of application of ME in these animals may not be the same that is required in humans. Last, we examined two groups of rabbits which had the “standard” electrocautery surgical management; there was no true control group which had no electrocautery. Therefore we did not study the effect of tissue handling and dissection on the inflammatory process. Either a sham group without appendectomy could have been use or appendectomy performed without electrocautery.

## Conclusion

To our knowledge, this study is the first real-time investigation of thermal spread produced by a monopolar coagulator during appendectomy. The obtained results suggest that the key factor leading to injury due to lateral thermal spread is duration of ME application, and complications might occur not only when the maximum power is applied (which is what the majority of such studies focus on) but also under certain conditions when a surgeon uses standard ME settings.

The spread of heat generated by ME through tubular anatomic structures can cause thermal injury of the intestinal wall in the regions distant to the surgical site, its necrosis and even perforation. Despite certain limitations of this study, the observed effects of monopolar coagulation can bear clinical relevance and require further investigation.

## Data Availability

The experimental surgery videos, thermography visualization, histology and MPO data used to support the findings of this study are available from the corresponding author upon request.

## Abbreviations

ME: Monopolar energy
MPO: Myeloperoxidase
ESU: Electrosurgical unit
LP group: Low power
SP group: Standard power
SD: Standard deviations
WSES: World Society of Emergency Surgery
(US) shears: Ultrasound
